# Hard of hearing learners in a school for the deaf: A case study in the Eastern Cape province

**DOI:** 10.4102/sajcd.v72i1.1089

**Published:** 2025-06-13

**Authors:** Thabisa P. Ndwandwe, Lavanithum Joseph

**Affiliations:** 1 Discipline of Audiology, School of Health Sciences, University of KwaZulu-Natal, Durban, South Africa

**Keywords:** d/Deaf, hard of hearing, function, Deaf Culture, South African Sign language, learners, communication, special school

## Abstract

**Background:**

Learners who are hard of hearing (HoH) in the Eastern Cape typically attend special schools for the deaf. Failures in mainstream schools result in learners being placed at a special school where South African Sign Language (SASL) is used as the medium of instruction. This is despite learners having residual hearing compared to their d/Deaf peers. This scenario is common in South Africa and often poses a dilemma for professionals in the field.

**Objectives:**

The objectives of this study were to describe the contextual factors of the audiological history (cause of hearing loss, age of diagnosis and use of amplification) and the communicative function of learners, to explore the school experiences of HoH learners in terms of their classroom and social experiences and to describe learners’ views on self-identity.

**Method:**

The study participants consisted of two groups, learners (eight) and their parents or caregivers (six). A case study design was used. The data sources for the study included participant interviews, direct observations and a review of records. Thematic analysis and descriptive statistics were used for data analysis.

**Results:**

The learners used SASL and had positive school experiences. They could communicate effectively in SASL and identified with the school community and Deaf Culture. Learners used speech-reading and some oral language. Parents and caregivers could not communicate in SASL with their children.

**Conclusion:**

Learners who are HoH had a sense of belonging in a school for the deaf where SASL is used as a medium of instruction.

**Contribution:**

The findings contribute to the understanding of learners who are HoH in the South African Special Needs Education context where research of this nature is lacking.

## Introduction

The term d/Deaf (Paul & Whitelaw, 2011) is used in the article to avoid assumptions about identity, and to include both audiologically deaf and culturally Deaf learners in the special school. The term Deaf is used to refer to individuals who identify or associate with the Deaf culture (South African National Deaf Association, 2018). The term deaf will be used to refer to individuals with a profound hearing loss who often communicate using sign language (WHO, 2020). The educational environment has a strong influence on a young learner’s social development and communication. The educational environment plays a vital role in influencing various aspects of a learner’s life (Roksandic et al., 2018). The social development and educational achievements of a child could have a significant influence on a learner’s transitions in life, from a school environment to a work or a higher education setting. The social, emotional and cultural needs of a child with hearing loss have to be considered when selecting a school; this includes considering the opportunity for interaction and communication with peers (Marschark, 2007).

The diagnosis of a child with a hearing loss, particularly one of school-going age, needs due consideration to be given to the communicative implications of the hearing loss as well as the school placement options available. Under the umbrella of aural rehabilitation services, services such as educational planning and classroom accommodations may be provided by audiologists (Tye-Murray, 2024). Audiologists play a particularly significant role in helping families, and children meet their audiological and educational needs through initial informational counselling. The selection of an appropriate school for a child with a hearing loss is often a critical decision for parents and families. Various factors need to be considered. In the South African context (where there is a lack of resources), it is evident that the choice of school placement is often not much of a choice but rather is dictated by circumstance. Research suggests that despite the advocacy for Inclusive Education for children with disability, approximately 70% of school-going children do not attend school, and of those in the school system, the majority are still placed in separate special schools (Donohue & Bornman, 2014). With only four public special schools available for learners who are deaf in the Eastern Cape, it is common for learners who are hard of hearing (HoH) to be placed in a special educational needs school, where South African Sign Language (SASL) is taught and practised within a Bilingual-Bicultural educational approach.

The White Paper 6 of 2001 (Department of Basic Education [DBE], 2010) advocates for learners with minimal learning barriers (regardless of their disability) to be educated in an inclusive educational setting, that is, with their peers – as opposed to being isolated in a separate school. This includes those children who are HoH or who have mild hearing loss and are fitted with adequate rehabilitative technologies such as hearing aids. However, learners who are HoH continue to be placed in special schools for learners who are deaf for various reasons, which include late diagnosis, poor access to aural rehabilitation services, lack of resources (especially in rural areas) and challenges within the Department of Basic Education.

The differences between the audiological and communicative functioning of children who are deaf versus those who are HoH deem it necessary to investigate the experiences of learners who are HoH in the context of a special school for the deaf that uses SASL as a medium of instruction. The information derived from a study of this nature had the potential to reveal the challenges, successes and support needs of learners who are HoH in a school for the deaf. Understanding the support needs of these learners was important to ensure learners functioned well and were able to thrive and achieve their academic goals in a school environment that may have often not been the first obvious choice for them. The findings of this research had the potential to also raise awareness of the adequacy or inadequacies of the current support systems provided for learners who are HoH in a special school for the deaf.

## Literature review

In South Africa, the education options available to learners who are deaf or hard of hearing (DHH) include Special Needs Education and Inclusive Education. Special Needs Education is the first form of schooling that was available for learners who are DHH (Aarons & Akach, 2002) with Inclusive Education being relatively more recent. Special Needs Education may be positive for learners as in such a school setting, children with significant permanent childhood hearing loss (PCHL) get an opportunity to interact with other DHH peers. In this school set-up, learners come to learn what they have in common with other d/Deaf individuals and are able to create bonds with one another (Aarons & Akach, 2002). These schools create an opportunity for d/Deaf individuals to socialise and form bonds. Attending a special school may benefit DHH learners, as DHH learners who are enrolled at these schools tend to be better adjusted and more emotionally mature (Marschark, 2007).

The Bilingual-Bicultural approach to teaching and learning is widely practised in many special schools for the deaf in South Africa (Akach, 2010). The approach promotes sign language as the main language of communication, and a spoken language is taught in its written form (Akach, 2010). The placement of learners who are HoH in a special school for the deaf exposes them to these practices. This may be unplanned or unknown at the time of enrolment by the parents or caregivers. Schools that use the Bilingual-Bicultural approach promote sign language as the main language of communication. Sign bilingualism, as a philosophy, does not exclude the development of speech (if possible) while promoting the development of spoken language in its written form (Akach, 2010). Proponents of the Bilingual-Bicultural approach believe that all children, despite their degree of hearing loss, stand to benefit from this approach (Akach, 2010). Learners who attend Bilingual-Bicultural programmes tend to develop functional skills in two languages and achieve greater self-esteem and confidence because of their having a healthy view of deafness and being in an environment that is accepting of them (Akach, 2010). This paper intends to address the topic of learners who are HoH in relation to the cultural, social and language practices in a special school in a transparent manner without taking a particular stance on the matter.

Studies have shown that DHH learners have a more negative self-concept compared to their hearing peers, in terms of communication and social competence – because of the developmental delays that result from early language deprivation (Mekonnen et al., 2016). This is typically associated with late diagnosis. Factors affecting self-concept in DHH learners may include poor parental communication skills, inadequate maternal bonding, social isolation, negative attitudes faced by these learners and rejection from family members and society at large (Mekonnen et al., 2016). The findings of these researchers indicated that DHH learners in special schools obtained higher scores for self-concept than those learners in special classes, in mainstream schools (Mekonnen et al., 2016). The results suggested that DHH learners in special schools had a higher and more positive concept of self when compared to DHH learners in other educational settings.

A school for the deaf that uses SASL for communication is often an unfamiliar environment for children who are HoH, who typically come from homes with families who are hearing. The same can be true for learners with sudden loss of hearing or acquired hearing loss as with cases such as chronic otitis media, who have been attending mainstream schools with hearing peers. It was therefore important to understand the views and experiences of learners who are HoH and to understand how these learners function in this environment. The research findings would be valuable in identifying the factors that influence and contribute to the learners’ views and attitudes. This could have implications for the nature of the educational and rehabilitative support needs of these learners. This study was therefore conducted with the hope that through its findings, it would provide information to parents, caregivers and other relevant stakeholders and decision-makers to make informed decisions regarding school placement for learners who are HoH.

The research question posed was ‘How do learners, who are HoH, function with regard to communication in a school for the deaf that uses SASL as a medium of instruction?’ The study intended to investigate how the learners coped with communication, their communicative traits and their ability to adapt to the language of communication in the school.

The aim of the study was to explore and describe learners’ audiological, communicative and socio-emotional functioning within a school for the deaf, where SASL was used as a medium of instruction. The study focused on three objectives, namely to describe the contextual factors in terms of learners’ audiological and communicative functioning, to explore the school experiences of the learners in terms of classroom and social experiences and to explore the views of the learners with regard to self-identity.

## Research methods and design

This study made use of a case study design. This study was exploratory in nature.

### Study site

This study was conducted at a school for the deaf that caters for DHH learners. The school was situated in Qonce, in the Eastern Cape and had a total of 200 learners, who originated from various parts of the Eastern Cape.

### Sampling

A non-probability convenience sampling method was used to select the study site as the site was conveniently located for the researcher. Purposive sampling was used to recruit participants based on the selection criteria, their availability and willingness to participate (Gravetter & Forzano, 2012). The selection criteria used included an exclusion and inclusion criteria. The inclusion criteria stipulated that learners from grades 6 to 12 could participate; they must have been enrolled at the school for a minimum of 6 months to ensure they had some significant exposure to the school environment, and they needed to have an existing audiology file at the school. The researcher relied on the available audiograms in learners’ files to be able to identify those learners who were HoH versus those who had more severe hearing loss. The selection criteria for parents or caregivers focused on the parents’ language proficiency. Parents or caregivers had to be proficient in English or isiXhosa to be able to participate in the study. The exclusion criteria excluded learner participants who had observable intellectual or physical difficulties that could potentially limit their participation in the study.

### Description of study participants

This study consisted of two groups of participants, learners who were HoH and their parents or caregivers. A total number of eight learners were used for the study. Learner participants included male and female learners from grades 6 to 12 who resided at the hostel on the school premises. The learner participants were ideally located and easily accessible. Table 1 shows that the age range of learners was 13 to 19 years, with grades ranging from 7 to 12. The mean age of enrolment at a school for the deaf was 7.3 years. Three participants (P1, P4 and P8) were multiple-grade repeaters and subsequently had a higher number of years enrolled at the school. A majority (five out of eight) of the participants were female.

**TABLE 1:** Demographic information of learners (*N* = 8).

Participant	Gender	Age (years)	Age of diagnosis (years)	Grade	Age of enrolment at current school (years)	Number of years in school
P1	Male	19	2	11	10	10
P2	Male	16	8	7	10	5
P3	Female	19	Unknown	12	Unknown	3
P4	Female	19	Unknown	12	Unknown	11
P5	Female	15	6	9	6	8
P6	Female	15	5	7	9	7
P7	Male	13	2	7	4	9
P8	Female	17	3	10	5	12

Note: Information retrieved from the audiograms in the learners’ files indicated that learners’ hearing thresholds ranged from mild to profound for the majority of participants, and the pure-tone averages for learners ranged from 48.3 dB to 80 dB in the better ear. This fell between the thresholds of 26 dB and 80 dB classified as HoH by the World Health Organization (2020). The type of hearing loss was unknown as there were no results for bone conduction. Speech audiometry results were also not available in learners’ files. Please see full reference list of this article for more information: Ndwandwe, T.P., & Joseph, L. (2025). Hard of hearing learners in a school for the deaf: A case study in the Eastern Cape province. *South African Journal of Communication Disorders, 72*(1), a1089. https://doi.org/10.4102/sajcd.v72i1.1089.

### Data collection instruments

The data collection tools used in this study included a researcher-developed interview schedule for learner participants, a self-administered rating scale for learners developed by the researcher, an observation schedule of spoken language adapted from Prutting and Kirchner (1987) and a review of the audiological records in the learners’ school files. A hardcopy (researcher-developed) form was used to record the information from the file reviews. A structured questionnaire was used for the telephonic interviews with parents or caregivers. A smartphone was used to capture the audio-visual data with the learner participants.

### Data collection process

A pilot study was conducted with seven learners with a similar profile before commencing data collection for the main study. For the main study data collection, the learner participants were invited to participate in a one-on-one, semi-structured interview of approximately 30 min with the researcher. A structured interview schedule was used. The researcher posed questions in both English and in the local language, isiXhosa. The researcher probed participants whenever it was deemed necessary. The interview sessions were video recorded using a smartphone. Audio-visual recording was used to capture the visual information in SASL while capturing the audio from the voicing-over of the SASL interpreter simultaneously. An SASL interpreter was used to facilitate the communication between the researcher and the learners during the data collection process. Once the interview was concluded, the learners were then required to complete a self-administered rating scale. An observation schedule was also used to note the researcher’s observations during an informal conversation with each participant. Once the data collection was completed with learners, the researcher proceeded to the phase involving parents or caregivers. The researcher contacted parents or caregivers via telephone and conducted a telephonic interview. A researcher-administered questionnaire was used during the interview. The questionnaire was administered in the language of choice (isiXhosa or English) of the parent or caregiver. Responses of parents or caregivers were captured on a hardcopy form developed by the researcher. The audio-visual data collected in this study were transcribed and analysed using thematic analysis. The quantitative data (hearing thresholds and participants’ demographic information) were analysed using the SPSS Version 27 for descriptive statistics.

### Ethical considerations

All ethical principles and guidelines were adhered to during the course of the study. Prior to commencing the research process, ethical clearance was obtained from the Biomedical Research Ethics Committee of the University of KwaZulu-Natal (reference number BREC/00002598/2021). Following full ethical clearance, permission to conduct the study was also obtained from the Eastern Cape Department of Education, the relevant school district and the school principal. Written consent was obtained from learners who were 18 years or older. Written parental consent was obtained for learners who were below the age of 18 years, together with written assent from these learners. Verbal consent was obtained from parent or caregiver participants for their participation as they resided far from the school. The audio-visual data collected using a smartphone were transferred to a password-locked laptop. The data were then transcribed using a laptop computer and stored. Data collected in hardcopy format were safely stored in a locked cupboard. Data collected in the study will be kept safe for 5 years. Thereafter, the data will be destroyed by permanently deleting digital files and shredding hardcopy paper data.

## Results

The researcher made use of a process of triangulation of results to report on all the findings from the different data sources used in the study. Qualitative data were categorised and analysed under the themes, such as auditory functioning, communicative functioning, school experience and self-identity.

### Contextual factors of audiological history and communicative functioning

#### Auditory functioning

The results of this study revealed that all the learners had a history of undergoing audiological assessments. All the learners had been in the past, fitted with hearing aids. None of the learners, however, were using their hearing aid device(s) at the time of the study. Hearing aids were reported as lost or damaged. The average age of diagnosis of hearing loss was 4.3 years. The hearing thresholds of learners ranged from mild to profound for the majority of participants with the pure-tone average (PTA) ranging between 48.3 dB and 103.3 dB for both ears. The PTAs for the better ear ranged from 48.3 dB to 80 dB, falling within the range of HoH.

The study attempted to describe a set of typical qualities that characterised the audiological profile of learners who are HoH. The results, as seen in Table 3, indicated that learners who are HoH typically presented with hearing thresholds ranging from a mild to a severe or profound range (30 dB to 115 dB) according to the configuration on the audiograms. Results derived from learners’ interviews also revealed that learners had access to sounds including music at loud levels, people’s voices at loud levels and hooting of motor vehicles:

‘I can hear a car when it hoots, I cannot hear a cat. If there are many cars, I can hear the sounds of the cars moving.’ (P1, Male, 19)

‘I can hear when people are speaking loudly.’ (P2, Male, 16)

‘I can hear sound on the floor, when somebody is whistling, or someone is calling loud.’ (P7, Male, 13)

Congenital hearing loss was commonly reported. A total of eight (100%) learners reported a history of using a hearing aid or amplification device in the past. Although learners reported a generally positive experience with using amplification as it ‘helped them communicate better’, none of them were actually using any form of an amplification device at the time of the study:

‘Yes I have in the past.’ (P1, Male, 19)

‘Yes in the past when I was young but now it got lost.’ (P2, Male, 16)

‘In the past, but now I don’t have.’ (P3, Female, 19)

#### Communicative functioning

The following information was obtained from the learners’ reports during the interviews. All the participants used oral communication as well as SASL. Learners employed different strategies when communicating with different communication partners, for example, when communicating with hearing individuals, they made use of speech-reading, spoken language and some SASL, but when communicating with d/Deaf peers, they used SASL with no verbal utterances. The findings revealed that all of the learners had spoken language (isiXhosa) and were able to communicate in spoken language. Learners could respond to speech at normal conversational levels, mainly because of their reliance on speech-reading. These learners typically had access to some sounds and found that wearing hearing aids helped them to access even more speech sounds, which helped them to communicate better with their hearing communication partners.

Spoken language was typically used in the home environment, combined with some signing and use of speech-reading. Interviews with the families revealed that parents could not communicate in SASL. Five of the learners reported that they sometimes experienced miscommunication with their families. When engaged in conversation, the researcher observed that five of the learners opted to use a combination of SASL with voice or mouthing of words. The minority, three, chose to use SASL only without voice. When engaged in conversation, all learners (eight) ‘always’ had appropriate use of oral language landmarks of conversation in terms of turn-taking, use of gestures, facial expressions and eye gaze during a conversation. However, only some had appropriate use of vocal quality (one) and intensity (three). Participants had varying levels of speech intelligibility, with all displaying speech errors to some extent.

### School experiences of learners

The study results, as indicated in Table 1, revealed that learners who are HoH were enrolled at a school for the deaf at various ages and indicate a trend of earlier admission for learners with more severe hearing loss (in conjunction with Table 3) compared to those with milder hearing loss. Parents and caregivers were commonly assisted by the Department of Education in their districts to enrol their children in a special school. The results revealed that learners generally had positive feelings towards their school environment. The learners expressed feelings of being content and free:

‘I’m happy. I have peace. I’m not worried.’ (P5, Female, 15)

Learners who had previously been in a mainstream school reported a more positive experience in a school for the deaf compared to a mainstream school. The participants described their experience of being in a mainstream school as unpleasant, as they experienced bullying, struggled to cope, and felt that they were different from other learners in the school:

‘Yes, but when I was young in the past, I went to a school for the hearing, but I realised I was deaf then I came to this school. When the hearing speak, I didn’t understand at the school for hearing people.’ (P1, Male, 19)

‘They would make wrong comments and laugh at my voice and words I would say incorrectly.’ (P5, Female, 15)

‘At the school for hearing, when I’m sitting with the other children, maybe the teacher is asking a word or a story, when I stand there, I feel the comments, the teacher will look at me, the other children I feel they laugh at me they comment about the way I read so the hearing school, it was bad for me.’ (P5, Female, 15)

All eight learners reported that they felt the school was fitting for them and that they were able to participate in all the school activities. The results of the study also revealed that participants were content in a school environment where SASL is used as a medium of instruction. It was concluded that learners in this environment were satisfied and had a positive school experience.

The learner participants listed issues such as the lack of technologies for teaching and learning, incidents of gossiping as well as a lack of extracurricular activities in the special school as some of the challenges that they experienced in the school. One learner reported communication challenges with the teachers, as teachers were not always fluent in SASL:

‘I feel good, but it’s hard when the teachers don’t know sign, sometimes I am stuck on how to help because the hearing is not there, but I try to lip read and ask them to speak slow so I can help them with sign language.’ (P4, Female, 19)

With regard to communication in the school environment, four (50%) of the learners reported that they ‘never’ experienced miscommunication with their friends at school. Three (37.5%) learners reported that they experienced miscommunication with their teachers at school. All eight (100%) learners responded positively when asked about their experience of learning in SASL. Learners reported that they understood the classwork when it was taught in SASL. However, two (25%) learners reported that they experienced some challenges in the classroom because of the teachers having difficulty communicating in SASL.

Based on participants’ reports and observation, it was determined that learners were fluent in SASL. All the participants reported that they had developed skills in communicating in SASL and could ‘communicate well in SASL’. Sign language was mainly learnt through emulating other d/Deaf learners in the school or through teaching by d/Deaf adults in the school. Four (50%) of the learners reported that they used SASL most of the time for communication. Five (62.5%) learners reported that they sometimes used speech or oral language to communicate with their teachers, peers and family members. The findings of the study further indicated that the learners could identify as d/Deaf despite their audiological results that categorise them as HoH. Results also indicated that these learners strongly identified with the Deaf Culture.

### Self-identity

The study findings revealed five (62.5%) learners identified as ‘hard of hearing’, while three (37.5%) identified as ‘d/Deaf’ (P 2, P5 and P7). Four (50%) learners felt that they were the same as everyone else in their school environment, while the other 50% reported that they felt different from other learners in the school, as the others were deaf and they themselves were ‘hard of hearing’ It was discovered that some of the learners chose to identify with the d/Deaf rather than the hearing, in solidarity with the Deaf community. The learners expressed that they felt they needed to ‘support’ the d/Deaf, fearing that they could be ostracised from the school community.

Results indicated that learners who are HoH had negative feelings and dissatisfaction with their interactions with people considered to have normal hearing sensitivity and who did not know SASL. The learners felt uncomfortable in their interactions mainly because of unrealistic demands and expectations for them to speech-read and understand conversation at normal conversational levels with their hearing communication partners:

‘It is difficult with the hearing, it is better with the deaf, I feel free.’ (P5, Female, 15)

‘They expect me to lip read perfectly, but I can’t, I’m not perfect.’ (P3, Female, 19)

The learners typically made use of some spoken language and relied on speech-reading and writing to communicate with communication partners who did not know SASL. Results revealed that these learners generally had negative attitudes towards interactions with people who are typically hearing or who do not know SASL. Learners reported that they felt ‘sad’ or ‘bad’ when interacting with hearing people. Learners reported a much more positive experience when communicating with people who are d/Deaf or know SASL. The learners reported that they felt ‘happy’ and ‘comfortable’.

## Discussion

The characteristics of learners who are HoH differ in terms of their abilities to listen and communicate, as learners who are HoH have a unique set of qualities that characterise them. The lack of use of amplification with learners who are HoH, who have some residual hearing was concerning and raises issues with the topic of fitting hearing aids for learners who attend a special school that uses SASL as a medium of instruction. It was discovered in this study that the majority of learners who are HoH had hearing loss that ranged from mild to profound range, with PTAs between 48.3 dB and 103 dB when using a three-frequency (500 Hz, 1000 Hz and 2000 Hz) average method of PTA calculation. No speech audiometry results were available for the learner participants. However, all the learners in the study had spoken language that was intelligible to some degree. Learners were also able to cope with conversation, mostly by relying on the use of speech-reading. It may have been interesting to assess the PTA correlation with the speech results to gain more details about the amount of speech information individual learners can access. Perhaps future research should be conducted to further investigate this. There is a need to further investigate the concept of ‘hard of hearing’ and possibly expand the definition to include individuals who have mild-to-profound hearing loss where two or three frequencies are in the profound range.

Learners who are HoH generally had a good experience in a school for the deaf that uses SASL as a medium of instruction. This was evident in learners’ positive responses and views concerning the school environment. A school for the deaf provided a more positive learning experience for the learners, as it is within this environment that they got the opportunity to interact with people with whom they shared a similar trait of hearing loss. Learners felt comfortable and felt a sense of belonging. It was concluded that learners who are HoH coped well in a school for the deaf as they could successfully interact with their immediate environment and participate actively in their environment without any fear of failed interactions. These findings were similar to those of Anglin-Jaffe (2020).

The participation of learners who are HoH in the deaf school environment was unrestricted as there were no social, communicative or acoustic barriers that existed for them in the school. There were, however, challenges that were experienced within the classroom situation as teachers were reported to be lacking proficiency in SASL.

This finding is similar to that of Ngobeni et al. (2020) who discovered that there were language barriers that existed in the classroom environment as teachers in a school for the deaf were not fluent in SASL. Ngobeni et al. (2020) reported that participants were unable to participate in the classroom as they were confused and unable to understand communication from the teachers who mostly used actions and gestures as opposed to SASL. It was discovered in this study that in some instances, the burden was put on learners who are HoH, who essentially had some access to auditory information and could also sign, to mitigate communication breakdowns that occurred in the classroom. Learners who are HoH sometimes had to assist in interpreting for the teacher and d/Deaf learners in the class.

Diaz (2014) studied the experiences of DHH students in mainstream education. The findings of this study revealed that DHH learners experienced difficulties in mainstream education. These difficulties were mostly a result of a language barrier that existed. It was found that DHH learners experienced negative feelings and feelings of loneliness when they were in school. Learners experienced rejection and felt that they were different from the other learners who could hear. The negative feelings lead to feelings of inferiority and poor self-esteem (Diaz, 2014). The results of that study concur with the results discovered in this current study where learners reported negative educational experiences in mainstream school and poor self-esteem because of bullying and a general feeling of being different, contrasted to the special school environment where learners felt comfortable and were able to identify with other learners through the shared trait of hearing loss.

Research suggests that the academic achievements of learners who are HoH in a bilingual environment are largely linked to the learners’ proficiency in sign language (Akmese & Acarlar, 2016). The more proficient the learner, the better their academic performance. This suggests that more emphasis should be put on training learners who are HoH in sign language when they are placed in a school for the deaf to improve their academic achievements. Proficiency in sign language could potentially ensure that learners who are HoH, who attend schools for the deaf that use sign language as a medium of instruction, are well integrated into their immediate environment. Lack of proficiency could lead to the risk of isolation and other negative effects on learners’ social and academic interactions within the school environment. Learners who are HoH may be considered multilingual as they are exposed to multiple languages. In the Eastern Cape, learners are typically exposed to isiXhosa in the home environment; SASL is used as the language of instruction at school and English as the language of writing. Research suggests that multilingual learners are often not proficient in any language. Baker and Scott (2016) suggested that learners need intensive language immersion to develop a strong first language base (L1).

These research findings suggested that when learners are not proficient or do not have a strong L1, there are often negative academic repercussions. Educational audiologists have a key role to play in being language mentors for learners and facilitating exposure and opportunities for learners to develop a strong L1 (SASL) proficiency. It may be beneficial for educational audiologists who are based in special schools to aid learners who are HoH in developing proficiency in the language of the school to help improve the academic and social outcomes of learners.

Although the acquisition and proficiency of SASL proved to be beneficial in the school environment, this was not the reality in the home environment. According to the results tabulated in Table 2, there was a discrepancy between the number of learners who use sign language versus the number of parents who can use sign language to communicate, suggesting that there are gaps in communication between learners and their parents or caregivers. The data suggested a possible gap in communication between families who are hearing and use a spoken language and their children who attend special school programmes and use sign language to communicate. In work conducted by Joseph and Alant (2000), exploring communication between mothers and their children with hearing loss who sign, it was evident that the lack of skills of mothers in sign language posed a barrier to communication between them and their children who are deaf. The study findings revealed that mothers were unable to communicate on an equal level with their children through SASL. The mothers’ lack of proficiency in sign language had serious implications for communication if sign language was used as the only language in communication. The information derived from this study and the past studies further supported and highlighted the need for professionals to continue engaging in a holistic, family-centred approach when providing intervention to children and their families. Family-centred practice guidelines stipulate that parents must be involved in all decision-making processes regarding the services that will be provided for their child and in setting goals and planning for services (Law, 2014). Intervention regimes and therapy programmes need to be planned in a holistic manner that best serves and benefits the needs of the child. More focus is needed in equipping audiologists with the skills and the information they need to be able to provide families with detailed information.

**TABLE 2:** Pure-tone audiometry results of participants (air conduction thresholds).

Participant	Air conduction threshold (decibel hearing level)							
	125 Hz	250 Hz	500 Hz	1 kHz	2 kHz	4 kHz	8 kHz	Pure-tone average
P1	R 75	75	75	65	65	70	70	68.3
	L 80	85	80	60	60	60	60	66.6
P2	R 45	40	55	55	70	85	55	60.0
	L 40	55	40	50	55	60	65	48.3
P3	R 55	70	70	80	80	75	65	76.6
	L 75	100	105	NR	NR	NR	NR	-
P4	R 50	50	55	85	90	105	NR	76.6
	L 45	45	65	100	NR	NR	NR	82.5
P5	R 45	60	60	70	60	55	60	63.3
	L 60	75	100	110	100	105	95	103.3
P6	R 35	50	80	95	105	NR	NR	93.3
	L 35	40	60	80	100	95	NR	80.0
P7	R 30	40	45	90	100	115	NR	78.3
	L 5	20	40	45	60	70	85	48.3
P8	R 40	55	60	80	85	95	90	75.0
	L 70	80	90	75	90	NR	90	85.0

Note: The parents or caregivers were included in the study to contribute information regarding the audiological and communicative history of the learners. A total number of six parents or caregivers participated in the study. Two parents who were recruited did not participate as they could not be reached telephonically at the time of the research data collection. The age of the parents or caregivers ranged from 34 years to 55 years. None of the parents knew how to communicate in SASL with their child. Information for two parents was unavailable, as they could not be reached telephonically.

R, thresholds for the right ear; L, thresholds for the Left ear; NR, no response (indicates that the patient did not respond to the tone stimulus at a particular sound/frequency level); SASL, South African Sign Language.

**TABLE 3:** Demographic information of parents or caregivers (*N* = 6).

Participant	Gender	Age (years)	Relationship to child	Use of SASL
PC1	Male	55	Father	No
PC2	Female	40	Mother	No
PC3	Female	43	Mother	No
PC4	Female	50	Mother	No
PC5	Female	34	Aunt	No
PC6	Female	40	Aunt	No

PC, Parent or caregiver; SASL, South African Sign Language.

Research suggests that 10% of parents who receive counselling are informed about all the communication options available, while only 4% are informed about more than one education methodology (Shezi & Joseph, 2021). This research revealed a gap in the informational counselling that parents received upon diagnosis of their child with a hearing loss, which may be indicative of the gaps in the knowledge of professionals working with these families. When parents or caregivers are given full information, they are able to make well-informed decisions and have realistic expectations. Families can be made aware of the bilingual option and continue to use both sign language and spoken language, provided aural rehabilitation and supporting technology are used. The possibility of learning more than one language and how these can coexist could be highlighted to parents or caregivers.

The idea of self is a dynamic concept that may be influenced by various factors in one’s environment. It is subject to change as an individual grows and experiences life. Different intrinsic and extrinsic factors influence an individual’s life. It is important for an individual to have a sense of self and identity. The Deaf Identity Development Model (Glickman & Carey, 1993) suggests that individuals with hearing loss choose to associate with either of the four cultural models, namely, the Hearing Identity, the Culturally Marginal Identity, the Immersion (Deaf) Identity and/or the Bicultural Identity (Goldblat & Most, 2018). Individuals with hearing loss, who identify with the Hearing Identity, perceive deafness as a medical pathology and view hearing as the standard for health and normality. The culturally marginal associates are those who feel that they do not belong to either the hearing or the deaf population and have difficulty immersing themselves in either society. Individuals who identify with the Immersion Culture have positive identification with Deaf people and use sign language. Individuals who identify with the Bicultural identity feel comfortable with both Deaf and hearing communities and may have a strong identification with the Deaf community, but they also value and feel comfortable with hearing people (Goldblat & Most, 2018).

According to the results of this study, it was concluded that learners usually had a more positive self-identity in the special school environment. Results revealed that learners who are HoH felt that they best fit in with people who are d/Deaf and find it difficult to associate and identify themselves with people who have normal hearing. It is important for learners to have a healthy sense of self as this may have a positive influence on outcomes in education, career and psychosocial development (Smolen & Paul, 2023). An individual’s self-identity is fluid and subject to change at any given point in his or her life. Intrapersonal factors related to deafness such as interactions with other DHH individuals, reactions to deafness, stigma and educational experiences may influence when and the extent to which an individual may identify as deaf, Deaf, HoH or a bicultural identity (Smolen & Paul, 2023). An individual may express multiple identities simultaneously at any point in their lives. It is important for audiologists and other professionals working with DHH individuals to be fully cognisant of the ever-changing and flexible nature of self-identity in individuals who are DHH so they are able to provide intervention programmes that are just as flexible in nature and respond to the immediate needs of the individual as they move through stages of life from school-going age, adolescence and adulthood. It is important for audiologists to acknowledge that self-identity is continually changing, and, therefore, interventions must be sensitive to these changes as opposed to a rigid, linear method of intervention. Families need to be made aware of this as well.

### Strengths and limitations

The use of multiple data sources allowed the researcher to access detailed information on the various aspects outlined in the objectives of the study and fulfil the aim of the study. This study was successful in providing some updated insight into the situation of learners who are HoH in a school for the deaf that uses SASL. The study findings revealed the communicative and audiological traits of learners who are HoH. Engagement with these learners provided the opportunity to explore and describe the views and opinions held by these learners with regard to them being educated in a school for the deaf with d/Deaf peers. There was limited information in this area of research in the Eastern Cape, as well as generally in the literature. This study was able to contribute to the field of knowledge.

The limitations of the study included the following:

- The study was only conducted in one special school for the deaf, located in the Eastern Cape. Conducting the study in more schools and in different regions would make results more generalisable.
- Access to learner participants was limited because of a high dropout rate experienced by the school and some prospective learners being on suspension as part of ongoing disciplinary processes.
- Two prospective parents or caregivers of participants could not be reached on their mobile devices because of network coverage issues.

### Study implications

The study implications are as follows:

- The study findings have implications on the theoretical understanding of learners who are HoH within educational settings.
- There is a clinical implication with regard to understanding the categorisation of hearing loss as ‘hard of hearing’ and the ability to access sounds through amplification and assistive technology.
- The importance of full diagnostic testing and reports in paediatric audiology, as critical educational decisions are made on audiological findings.
- The study findings have the potential to influence the process of selecting and acquiring school placement for learners who are HoH.
- The findings of the study highlight the need for further focus and investigation on learners who are HoH and the understanding of these learners’ support needs.
- The findings can be used to guide the support of HoH children in mainstream schools within an inclusive educational context.
- The findings of the study could potentially assist in informing programmes designed for upskilling of teachers for practice in both inclusive and special education schools including their use of SASL.

## Conclusion

Learners who are HoH had positive school experiences in a school for the deaf that used SASL as a medium of instruction. This was mainly because learners were able to participate in this environment successfully without any limitations. A school for the deaf created an environment where learners who are HoH could form meaningful social bonds and have successful interactions with their peers. The contextual factors in a school for the deaf gave identity and a sense of belonging to learners, as they could associate with people with whom they share a common trait – hearing loss. Some challenges existed in the classroom environment, as communication breakdowns occurred, mainly because the teachers were not proficient in SASL. The views on self-identity and self-concept of learners who are HoH discovered in this research challenge audiologists and other proffesionals working with people who have hearing loss, to be mindful in providing intervention that is sensitive and responds to the individual’s immediate needs as they navigate life transitions.
